# Medical Items and News of Interest to the Physician

**Published:** 1884-07

**Authors:** 


### For the Use of Physicians.

CULLED FROM THE STANDARD MEDICAL JOURNALS OF THE WORLD.

NOTE.—These formulæ are intended only for the regular practitioner of medicine, and should be employed only by him, or under his immediate supervision.

**Quinine in Excessive Sweating.**

Dissolve one drachm in a pint of alcohol and sponge the skin.—*Med. and Surg. Rep.*

**Helenine.**

Dr. Valenzuela (*Bull. Gen. de Thier.*) gives 1-6 gr., ten times daily, in broncho-pneumonia, tuberculosis, whooping cough and respiratory diseases. May be used by inhalation.—*Med. Annals.*

**Psoriasis.**

℞

Sodii arseniatis (pellets), gr. $\frac{1}{10}$.

Sig Dissolve in a little water, and inject into the subcutaneous tissue every day, the dose to be gradually increased until all the eruption disappears.—*Medical Bulletin.*

**Eczema.**

Bicarbonate of soda is recommended by Rorseau as an application in eczema. He uses it in the form of a pomade in the strength of ʒ j of the soda to ℥ j of lard or other base. He holds that it alters the morbid anatomical elements of the skin and restores it to the normal state. —*Columbus Medical Journal.*

**Antiseptic Inhalation.**

A lamp heats a metal tube coiled in a tin boiler; air passes into the free lower end, and from the free upper end of this tube is conducted hot through a receptacle filled with dry medicated tow, and through an inhaling tube with valves to the mouth.—*Med. and Surg. Reporter.*

**Effect of Pilocarpin Upon Color of the Hair.**

Dr. Pohlman says that he has succeeded in demonstrating the capability of pilocarpin to darken the color of the hair in the presence of pigmentary matter, perhaps by a stimulating action on its formation; but that the drug is unable to produce color where pigmentary matter is absent, as shown in the case of experiments upon albino rabbits.—*Buffalo Medical Journal.*

**Aconitia (Duquesnel's) in Facial Neuralgia.**

The dose at first is $\frac{1}{250}$ grain to be cautiously and gradually increased to $\frac{1}{80}$ grain, repeated three times a day. The effect of the remedy must be carefully watched.—*Medical Bulletin.*

**Adherent Placenta.**

Dr. J. Field, Kansas City, in six cases saved the woman by pumping cold water into the umbilical cord. One patient was in convulsions when the placenta came away.—*The Clinique.*

**Lee's Injection for Gonorrhœa.**

Copper sulphate, two grains; dissolve in water one-half fluiddrachm, and then add olive oil, one fluidounce; corbolic acid, sixteen grains: Magendie's solution, one-half fluiddrachm. Mix. Shake well, and inject a portion night and morning.—*Med. Bulletin.*

**Corrosive Sublimate in Dysentery.**

This remedy, in half minim doses of the liquor hydrargyri bichloridi of the British Pharmacopœia, administered hourly, speedily and infallibly cures dysentery (barring the tropical and infantile forms), according to Dr. March, in the *London Medical Times and Gazette.*

**Herpez Zoaster.**

This is a very satisfactory remedy for Herpes Zoaster:

℞

Morphinæ sulph. gr. $\frac{1}{4}$.
Atrophinæ sulph. gr. $\frac{1}{80}$.

M. Sig. Give as a hypodermic injection every three or four hours until pain is relieved.

**Erysipelas.**

The following is one of Dr. Shoemaker's favorite prescriptions for this disease:

℞

Glycerini. } āā ℥ ij.

Tinct. ferri chloridi }

M. Sig. Take from one to two teaspoonfuls in water every three hours until all local irritation subsides.

**Hot Water to the Ear.**

Dr. Strawbridge, Phila., poultices the external ear in the following ingenious manner: He lays the patient's head on a table and fills the external ear with as hot water as can be borne. Over the ear are applied towels soaked in very hot water, the surplus water being drained off by squeezing the soaked towels between dry ones.—*Med. Herald.*

**Cramp.**

A writer in the *British Medical Journal* says: The best remedy for cramp, the simplest and the most efficacious that I know of, is a band of cork It is easily made by cutting a small new wine-cork into thin slices, which must be sewn close together upon ribbon or tape an inch wide. It can be tied round any part affected, and worn during the night.

---

**Eczema (Acute).**

The two prescriptions below are excellent for acute and chronic eczema :

Zinci carbonatis }

Plumbi carbonatis }

Lac. sulphuris } āā ʒ i.

Pulveris marantæ }

Ungt. simplicis ℥ j.

Ft. Ungt. molle.

Sig. Pencil lightly over the face.

---

**Eczema Chronic.**

℞. Extract. malt }

Ol. morrhuæ } āā ℥ ij.

M. Sig. Two teaspoonfuls three times daily.
—*Medical Bulletin.*

---

**Chronic Tuberculosis.**

Creasote cures a small proportion of cases of chronic tuberculosis, but it is useless in tuberculosis florida. Prof. Bartholow vaporizes it with iodine by means of hot water (120°), and the patient inhales the vapor slowly and deeply from a distance of fifteen to twenty inches from a vaporizer; or, grs. iii–v are given in a pill with tolu, three or four times a day, the dose being gradually increased until the urine is darkened. Its action is thought to be on the bacillus, and is better than carbolic acid.—*College and Clinical Record.*

---

**Jameson's Liniment.**

Dr. P. H. Jameson, of Indianapolis, frequently prescribes a liniment, the formula of which is as follows:

Tincture of aconite, 1 oz.
" belladonna, 1 oz.
Soap liniment, 2 ozs.
Chloroform, 2 drs.

This, says the Indiana *Pharmacist*, from which we copy the formula, is known by many people as Jameson's Liniment, but is never prescribed by the worthy doctor under that name; he alwas writes the formula in full. It is an excellent remedy for bruises, sprains and neuralgic pains.

**Cholera Morbus.**

Prof. Da Costa recommends the following for the cramp of cholera morbus :—

℞.

Chloral. hydrat, ℥ j

Liniment. saponis. ℥ iv—vj. M.

Sig.—Apply with friction to the abdomen.

---

**Acute Eczema.**

Prof. Rohe in a paper upon acute eczema, recommends the following for constitutional treatment in that affection:

℞

Tr. ferri. chloridi ʒ ij.

Acidi phosphorici dil ʒ vi.

Spiriti limonis ʒ ii.

Syrupi ad ℥ vi.

M. Sig. A dessert spoonful every four hours.

---

**General or Constitutional Treatment in Diphtheria.**

℞

Saturated Tr. Phytolacca, Dec. gtts xx

Baptisia Tinct. gtts. xx.

Potassa chlorate ʒi.

Alcohol ʒ i.

Aqua Pur. ℥ iii.

M. et. Sig. One teaspoonful every one hour.
—*Med. Investigator.*

---

**Corrosive Sublimate.**

The antiseptic virtues of this drug were first made known by R. Koch. Tarnier employs it in his maternity hospital. Every attendant on entering the ward must wash the hands and arms in a solution of corrosive sublimate (one in one thousand). The patient's genitals are bathed in a solution of one part in two thousand; this is the strength for vaginal injection. Billroth employed it as a surgical dressing (one in five thousand) in a case of suspected anthrax. —*Medical Annals.*

---

**Caffeine in Heart Disease.**

Prof. Lepine claims that caffeine is as efficacious as digitalis in retarding the heart's action and in increasing its force. In comparing the relative merits of the two drugs he asserts—

1. It acts more rapidly than digitalis, and in fatty heart where the latter is contra-indicated, there is no doubt but that it does good.

2. It is tolerated better than digitalis.

3. Most patients prefer it to digitalis.

Where caffeine produces insomnia it is contra-indicated. To produce benefit the dose must be from 9 to 30 grains.—*Theraputic Gazette.*

**Treatment of Catarrhal Pneumonia.**

R.

Potas. bicarb ℈ij

Tinc. verat. vir. M xxiv.

Liq. ammon. acetat. ℥ iv.

M. Sig. tablespoonful every three hours.—From a paper read by J. W. C. CUDDY, M. D., and published in the *Med. Chronicle.*

**Santonine in Gleet.**

A writer in the *Lancet* states that he was treating a patient for lumbrici and gave him santonine. The patient returned delighted, saying, "You have not only killed the worms, but have cured my gleet." He has since used it with good effects. Five grains each of santonine and sugar of milk to be taken twice daily, fasting.

**A Wart Cure.**

The following is recommended by Vidal as another addition to the long but unsatisfactory list of remedies recommended for the removal of warts: He bandages the wart covered hands in flannel and green soap. After a number of such applications the warts are said to become soft, in which condition they can be easily removed.—*Therapeutic Gaz.*

**Bromide and Iodide of Sodium.**

Mr. T. J. Hudson, M. B., of Leeds, gives in the *Lancet* his experience of these salts, and compares them, as therapeutic agents, with the corresponding potassium compounds. The chief distinction is that the sodium compounds are far less depressant than those of potassium, have a pleasanter taste, and are less likely to occasion diarrhœa or other bad effects. Mr. Hudson has had opportunities of watching the effects of the drug in a number of cases.

**Nasal Polypi.**

Dr. William Ralph Bell, in the Canada *Medical Record*, describes a new painless and simple method of removing nasal polypi. His patient is instructed to blow strongly through the affected nostril while he closes the other with his finger. This brings the polypus up so it can be seen through the external nares. He then injects into the tumor with a hypodermic syringe fifteen or twenty minims of a solution of tannic acid in water, twenty grains to the fluid drachm. In a few days the tumor shrivels, dries up, and comes away without trouble or pain, the patient usually removing it with the fingers or by blowing the nose.—*Ind. Med. Record.*

**Quassia for Mosquito Bites.**

Decoction of quassia, applied to mosquito bites, constitutes an excellent remedy for the relief of the itching and irritation. When applied to the exposed portions of the body it is also a preservative against the attacks of these very disagreeable and annoying pests.—*Ther. Gaz.*

**Tonic Bitters.**

Gentian root, six ounces; bitter orange peel, five ounces; cassia bark, one ounce; chamomile flowers, one ounce; raisins, eight ounces; coriander seed, two ounces; caraway seed, one-half ounce; cardamon seed, one ounce; red saunders, four ounces; dilute alcohol, one gallon. Macerate two weeks and filter. Dose, two or three teaspoonfuls.—*Med. Bulletin.*

**Corrosive Chloride of Mercury in Diphtheria.**

To children of less than ten years are given from one-sixteenth to one-twelfth grain doses of the corrosive chloride, every four hours, until arrest of the diphtheritic process, which is, as a rule, after a few doses. If ptyalism occur, the remedy should be abandoned, but while there exists any diphtheritic virus in the system salivation is not apt to occur.—*Ther. Gazette.*

**The Prophylactic Value of Turpentine Vapor in Infectious Diseases.**

Dr. H. Valandt writes in the *Ugeskrift for Laeger*, vol. viii., No. 8, 1883, concerning the value of oil of turpentine iu the treatment and prophylaxis of diphtheria and the exanthematous diseases. He states that he has never seen any of these diseases spread from a sick child to the other members of the family when this remedy was employed. In many of his cases no isolation could be attempted, as the mother was the only female in the family and was obliged to take care of both the sick and the well, continually passing back and forth from one to the other. His method was to pour from twenty to forty drops of a mixture of equal parts of turpentine and carbolic acid into a kettle of water, which was kept simmering over a slow fire, so that the air of the sick room was constantly impregnated with the odor of these two substances. He claims also that by this means a favorable influence is exerted upon the exudation in diphtheria, although it is by no means curative of the disease, and should never be relied upon to the exclusion of other remedies.

**Anti-Galactagogue.**

To check the secretion of milk, Dr. Verral[1] (*British Medical Journal*) recommends iodide of potassium, eight grains, and quinine sulphate, twenty-three grains, three times a day.

**Puerperal Septicæmia.**

Iodoform, five drachms; gum arabic, starch and glycerine, each half a drachm. Make into three suppositories about an inch and a half in length. These are passed into the uterus by forceps. Iodoform adheres to wounds, is insoluble, of slow vaporization; hence it continues to act for a long time, renders unnecessary the frequent irritating disturbance of the parts, unavoidable in treatment by intra-uterine injections.—*Dr. W. Gardner and Dr. T. J. Alloway, Canada Med. and Surg. Journal.*

**Lobelia Enemas to Produce Emesis.**

Dr. J. S. Prettyman not long since communicated the following to the *Medical Record:*—"I was consulted as to what should be done in the case of an old man who had swallowed (or rather half swallowed) a peach-stone, which had lodged about the middle of the æsophagus. He could swallow nothing, and the tube seemed to be completely occluded. I advise rectal injections of lobelia inflata. The consultant, Dr. J. O. Pierce, accordingly administered ounce doses of the fluid extract every fifteen minutes until three ounces were thus used.

**Iodide of Potassium in Asthma**

The favorable observations of Germain See, Winternitz and others, with iodide of potash in asthma, have been verified. Frensdorf, who recommends it as a prompt and efficient remedy that cannot be compared with any other drug, employs it in this affection. He dispensed it in doses of 3 grammes (gr. xiv) three times daily. Not unfrequently the dyspnœa yielded the same day; if not, smaller doses were repeated the following day. The catarrhal condition that usually accompanies an attack was treated with 1.5–2.0 grammes (gr. xx.xxx) continued for some time. In the author's estimation this plan of treatment also did much good as a prophylactic, if not to keep them away entirely, then to lengthen the intervals between the attacks. Iodism when present is no reason why the remedy should be suspended ; the symptoms disappear very rapidly and the treatment can be continued.—*Mittheilungen des Vereins der Aerzte.*

**Remedy for Gonorrhœa.**

A correspondent of the *Chemist and Druggist* communicates the following, which he says is a popular remedy for gonorrhœa in Edinburgh, where it is known as *Nesbit's Specific:*

LIQUOR SANTAL FLAV. C. COPAIB. ET CUBEBA.

℞

Ol. santal. flav. ℥ v.

Ol. copaibæ. ℥ ss.

Ol. cubebæ. ℥ ss.

Ol. pimentæ. ʒ j.

Ol. cassiæ. ʒ j.

Spt. vini rect. ad. Oj.

Dose, ʒ j twice a day in water.

**Goiter—Fluoric Acid.**

Dr. Edward Woakes gives, in the *Lancet*, a detailed account of a number of cases of goiter cured by fluoric acid internally. He begins treatment with fifteen minims of a one-half per cent. dilution of the acid three times a day, and, if necessary, increases the dose to twenty, thirty, forty, or even seventy minims, and extends the time to several months. His results are quite remarkable, even in cases that had resisted iodine, bromine, iron, etc. In a few, it was conjoined with injections of tinct. iodine. Very few failed to be reasonably benefited, and in eighty-five per cent. the cure was decided.—*Louisville Medical News.*

**Remedies for Toothache.**

When the nerve is exposed in the carious cavity the following paste is used by dentists: Arsenious acid, 3 parts, sulphate of morphia; 2 parts, and sufficient creasote to make a paste. A minute quantity of this introduced into a cavity previously dried with absorbent cotton, and covered with a small plug of cotton moistened with collodion, will usually suffice to destroy the nerve. Care must be taken to prevent contact of this paste with the soft parts, as it causes a painful ulcer.

Dr. J. R. Irwin recommends, in the *N. C. Medical Journal*, for April, the chewing of cinnamon bark for the relief of toothache. He says that if the bark is of good quality the pain is immediately relieved; that it is equally efficacious with creasote, carbolic acid, etc., without their disagreeable features. It is certainly a simple and an agreeable remedy, and no reader who does not let this note slip from his memory, will fail to put it to the test when the occasion calls for it.—*Therapeutic Gazette.*

**The Ether Spray an Immediate Cure for Neuralgia.**

Dr. McColgan extols the value of the ether or rhigolene spray for the instantaneous relief, principally, of facial neuralgia. He first had occasion to observe its good effects upon his own person, he having suffered greatly from facial neuralgia. Since curing himself, he had occasion to test its efficiency in about twenty cases. The result was invariably a most gratifying success. In many instances a permanent cure was established. He attempts to explain its action by supposing a complete change to take place in the nutrition of the affected nerve in consequence of the intense cold acting as a revulsive.—*St. L. Med. Journal.*

---

**Iron in the Treatment of Skin Diseases.**

Dr. Cesarini has employed the perchloride of iron with advantage in a large number of chronic skin affections. He uses an ointment of from one to three grams of perchloride of iron to thirty grams of lard. He concludes from a number of observations that: 1, perchloride of iron (internally administered) is the most efficacious agent in the treatment of simple or hemorrhagic purpura; 2, it is very useful to combat the anæmis which often accompanies certain cutaneous affections, such as rupia, ecthyma, and impetigo; 3, its external employment gives excellent and speedy results in ulcers of scrofulous and syphilitic origin; 4, in the form of ointment it constitutes a good remedy in the squamous skin diseases, especially in psoriasis.—*Druggists' Circular.*

---

**Jequirity in Trachoma.**

Dr. E. P. Brewer, Norwich, Conn., treated a case of trachoma with jequirity, after five years of treatment had failed. Three applications of a one per cent. solution were made under and upon the lids each day for three days. On the third day, temperature 100 degrees, pulse 90 ; on eighth day granulations mostly absent.

The progress of the disease is described in detail up to the sixty-seventh day, when "the cornea is still hazy and one blood vessel is visible in its substance. Some thickening of the lid remains. The palpebral conjunctiva is smooth and vivid in appearance, daily losing the deep red hue. The ocular conjunctiva is normal in aspect, yet on slight exposure shows some evanescent injection." The doctor pronounces the case cured.—*Therapeutic Gazette.*

**Treatment of Gonorrhœal Opthalmia.**

Mr. Charles Bader, surgeon to Guy's Hospital, strongly recommends the use of the following ointment:

℞

Red oxide of mercury, one grain.
Daturine or etropine, one-fifth grain.
Vaseline, one ounce.

M.

The ointment is to be injected twice a day under the upper lid by means of a small glass syringe with a flat nozzle. The surrounding parts are to be thickly smeared with the ointment and the eyes bandaged. In three cases reported he obtained excellent results.—*Lancet.*

---

**The Influence of Spices on Digestion.**

Hasson has been making some experiments on this question, which he publishes in the *Wiener Med. Blat.* He has carried out his experiments with artificial gastric juice, in which he has placed meat which had lain for four days in wine, vinegar, oil, charcoal, and common salt respectively. The meat which had been immersed in white wine dissolved the most rapidly, that in vinegar, oil, and charcoal just as quickly as fresh meat, while the salt meat was much more difficult to dissolve. He then experimented on the influence of salt, and found that small quantities hastened digestion, but that a large amount retarded it. Acetic acid seemed to hasten digestion in exact proportion to the dose.—*Ther. Gaz.*

---

**Glycerine After Bathing.**

Like the ancient Romans, D. S. Troy, of Montgomery, Ala., has taken to using glycerine after bathing, with most happy results. He puts one drop of attar of roses in two ounces of glycerine, and rubs the body after bathing. The result was charming: it left the skin soft, with all of its functions in as full operation as before the bathing, and with a delicious sensation of perfect cleanliness. He has been using it for four or five years regularly, leading a life more sedentary than ever, and it seems to supply to the skin a vigor similar to that resulting from muscular exercise. It has been of great benefit to his general health and personal comfort. No care is required in rubbing it on, except to see that it is applied to all the skin except the face, and particularly to the soles of the feet. If any remains after the rubbing, it can be readily wiped off with a towel. —*Popular Science News.*

**Naphthol as a Cure for Itch.**

Guerin prepares a mixture by dissolving 10 parts of naphthol in five parts of ether and then mixing it with vaseline about 100 parts. This ointment is applied locally and may be used in all stages of scabies; when kept from the action of the air it will remain good for some time.—*Journal de Therap.*

**Treatment of Condylomata.**

Salicylic acid and boracic acid, says the *Med. Surg. Reporter*, are very good remedies in syphilitic condylomata.

Formerly we often used to remove larger warts of that kind with the scissors, and then cauterized the wound; but since we have been employing the following powder, which is dusted three times daily over the new growths we have never had occasion to have recourse to any other remedy:

℞

Hydrarg. muriat. mit., gr. xxx,
Acid. borac., gr. xv,
Acid., Salicylic, gr. v.
M. F. pulv.

All moisture and disagreeable odor at once ceases, and who has not seen the effect of this powder, would scarcely believe it; the condylomata visibly dwindles away.

**Carbolic Acid Injections in Hæmorrhoids.**

G. A. Smith in the *Therapeutic Gazette* gives the following prescriptions which he uses successfully in hæmorrhoids:

℞

Acidi carbolici,

Napthalini, aa ʒ j.

Morphinæ acetatis.

Hydrast, sulphatis, aa gr. j.

Aquæ dest. ʒ iij.

Mix. ft. sol.
Sig. Inject into tumor through the base every four days till cured.

As a local anæsthetic:—

℞

Etheris sulphurici, ℥ ij.

Camphoria, ℈ij.

Mix.

As an ointment:—

℞

Acidi tannici,

Hydrarg, chlorid, mitis, aa ʒ j.

Ol. lim.

Ol. origani aa ʒ ij.

M. Sig. Apply externally.

**The Prevention of Puerperal Fever.**

There has been a time when everything was vigorously scrubbed, no pictures adorned the walls, no carpet decked the floor, and carbolic acid was used without stint, but I have never observed that these precautions exerted the slightest influence in the prevention or the stamping out of puerperal fever.

The fumes of sulphurous acid have since been substituted for the old-time scrubbing with the best results, and I would strenuously recommend the modes of disinfection employed by the Board of Health in all cases where there had been previously scarlatina, diphtheria, typhoid or erysipelas in the household. In this connection, I would state that a woman never ought to be confined in the room adjacent to the bath-room, as I believe that puerperal women are extremely sensitive to sewer gas poisons.—*Prof. Wm. T. Lusk, in New York Medical Journal.*

**Remedy for Ear Ache.**

A correspondent of the *Druggists Circular* submits the following, which he claims, after repeated trials, has never failed to afford almost instant relief: Olive oil, one drachm; chloroform, one ounce; mix and shake well, then pour twenty-five or thirty drops into the ear and close it up with a piece of cotton, to exclude the air and retain the moisture.

While this combination may do all that this correspondent claims for it, we should, nevertheless, suggest that it be administered very tentatively. It is possible that the olive oil may modify the action of chloroform thus dropped into the ear, and prevented from evaporating with a piece of raw cotton, although we should be inclined to be extremely careful in the use of this combination. We can recommend, from our personal experience, a certainly not less effectual means of administering chloroform, and one which is absolutely devoid of danger. This is to loosely fill the bowl of a common clay pipe with cotton batting, upon which pour as much chloroform as it will retain without dripping. This done insert the end of the stem carefully into the ear, and placing the opening of the bowl in the mouth blow gently the vapor of chloroform against the tympanum. We have found this to be an exceedingly effectual relief for the earache of children, uncomplicated, of course, with inflammatory disturbances.—*Ther. Gaz.*

